# Are Strength Indicators and Skin Temperature Affected by the Type of Warm-Up in Paralympic Powerlifting Athletes?

**DOI:** 10.3390/healthcare9080923

**Published:** 2021-07-22

**Authors:** Marcelo de Aquino Resende, Felipe J. Aidar, Roberta Barreto Vasconcelos Resende, Gracielle Costa Reis, Layanne de Oliveira Barros, Dihogo Gama de Matos, Anderson Carlos Marçal, Paulo Francisco de Almeida-Neto, Alfonso López Díaz-de-Durana, María Merino-Fernández, José Vilaça-Alves, Breno Guilherme de Araújo Tinoco Cabral, Eduardo Borba Neves, Victor Machado Reis, Filipe Manuel Clemente, Nuno Domingos Garrido

**Affiliations:** 1 Department of Physical Education, Tiradentes University (UNIT), Aracaju 49010-390, Brazil; marcelo.aquino@souunit.com.br (M.d.A.R.); roberta.resende@souunit.com.br (R.B.V.R.); gracielle.reis@souunit.com.br (G.C.R.); 2 Group of Studies and Research of Performance, Sport, Health and Paralympic Sports (GEPEPS), Federal University of Sergipe (UFS), Sao Cristovao 49100-000, Brazil; fjaidar@gmail.com (F.J.A.); layanneoliveira@cepiexpansao.com.br (L.d.O.B.); dematosd@myumanitoba.ca (D.G.d.M.); acmarcal@yahoo.com.br (A.C.M.); 3 Program of Physical Education, Federal University of Sergipe (UFS), Sao Cristovao 49100-000, Brazil; 4 Program of Physiological Science, Federal University of Sergipe (UFS), Sao Cristovao 49100-000, Brazil; 5 Department of Physical Education, Federal University of Sergipe (UFS), Sao Cristovao 49100-000, Brazil; 6 Cardiovascular & Physiology of Exercise Laboratory, University of Manitoba, Winnipeg, MB R3T 2N2, Canada; 7 Department of Physical Education, Federal University of Rio Grande do Norte (UFRN), Natal 59078-970, Brazil; paulo220911@hotmail.com (P.F.d.A.-N.); brenotcabral@gmail.com (B.G.d.A.T.C.); 8 Sports Department, Physical Activity and Sports Faculty-INEF, Universidad Politécnica de Madrid, 28040 Madrid, Spain; alfonso.lopez@upm.es; 9 Faculty of Health Sciences, Universidad Francisco de Vitoria (UFV), 28223 Madrid, Spain; m.merino.prof@ufv.es; 10 Research Center in Sports Sciences, Health Sciences and Human Development (CIDESD), Trás os Montes and Alto Douro University, 5001-801 Vila Real, Portugal; josevilaca@utad.pt (J.V.-A.); vmreis@utad.pt (V.M.R.); 11 Universidade Tecnológica Federal do Paraná (UTFPR), Curitiba 80230-901, Brazil; eduardoneves@utfpr.edu.br; 12 Escola Superior Desporto e Lazer, Instituto Politécnico de Viana do Castelo, Rua Escola Industrial e Comercial de Nun’Álvares, 4900-347 Viana do Castelo, Portugal; filipe.clemente5@gmail.com; 13 Instituto de Telecomunicações, Delegação da Coilhã, 1049-001 Lisboa, Portugal

**Keywords:** warm-up, muscle force, performance, resistance training, thermal imaging, physiology

## Abstract

(1) Background: the present study aimed to evaluate the effect of different types of warm-ups on the strength and skin temperature of Paralympic powerlifting athletes. (2) Methods: the participants were 15 male Paralympic powerlifting athletes. The effects of three different types of warm-up (without warm-up (WW), traditional warm-up (TW), or stretching warm-up (SW)) were analyzed on static and dynamic strength tests as well as in the skin temperature, which was monitored by thermal imaging. (3) Results: no differences in the dynamic and static indicators of the force were shown in relation to the different types of warm-ups. No significant differences were found in relation to peak torque (*p* = 0.055, F = 4.560, η2p = 0.246 medium effect), and one-repetition maximum (*p* = 0.139, F = 3.191, η2p = 0.186, medium effect) between the different types of warm-ups. In the thermographic analysis, there was a significant difference only in the pectoral muscle clavicular portion between the TW (33.04 ± 0.71 °C) and the WW (32.51 ± 0.74 °C) (*p* = 0.038). The TW method also presented slightly higher values than the SW and WW in the pectoral muscles sternal portion and the deltoid anterior portion, but with *p*-value > 0.05. (4) Conclusions: the types of warm-ups studied do not seem to interfere with the performance of Paralympic Powerlifting athletes. However, the thermal images showed that traditional warm-up best meets the objectives expected for this preparation phase.

## 1. Introduction

The warm-up was identified as essential to maximize the athlete’s performance in different sports and physical activities [1]. When addressing performance in adapted sports and Paralympic powerlifting, the warm-up has also been presented as a determinant for performance [1]. Warm-ups aim to improve nerve conduction, combined with an increase in temperature [2,3,4]. Specific warm-ups have been shown to improve strength [5,6,7], however, variations in the type of warm-up can be harmful [8].

The warm-up performed before training and competitions seems to increase body temperature, providing a decrease in stiffness, an increase in the rate of nerve conduction and an increase in metabolic efficiency [2,3], an improvement in muscle strength and power production [9], and an improvement in mechanical efficiency and contraction speed [10,11]. In this sense, it seems that during short-term static stretching, neuro-muscular activation and muscle-tendon stiffness seem to be unaffected. Among other factors, this may be due to an elevated muscle temperature, which tends to lead to an increase in the speed of conduction of muscle fibers and a better binding of contractile proteins (actin, myosin) [12,13], and stretching can provide this improvement.

On the other hand, with specific warm-ups with higher loads, the capacity to produce force tends to increase [14], that is, the force tends to improve [7]. A specific warm-up tends to increase strength, improving efficiency, training intensity, with improvements in strength and velocity [14,15,16]. However, variations in the type of warm-up can be harmful [8]. Currently, there is no consensus between the effect of different types of warm-ups [17,18,19], and the indications of the types of warm-ups are still very controversial [7]. Thus, the study raised the following hypotheses regarding strength and temperature: (i) traditional warm-up tends to be better; (ii) warm-up as stretching tends to be better; and (iii) there are no differences between the types of warm-up.

Therefore, the present study aimed to evaluate the effect of different types of warm-ups on the strength and skin temperature of Paralympic powerlifting athletes. It was hypothesized that the warm-up methods are not capable of altering the performance of Paralympic powerlifting athletes.

## 2. Materials and Methods

### 2.1. Sample

Fifteen male Paralympic powerlifters volunteered for this study. Every participant was a competitor involved in national competitions and was eligible for this sport under the International Paralympic Committee (IPC). All participants met the functional classification criteria of the IPC [20], with disabilities in the lower limbs, with the unique classification being legible and ineligible. Participants were required to have participated in a minimum of one competition at the national level over the past 12-month period and the average prior experience in the sport was 2.43 ± 1.03 years. The physical impairments of the participants were varied as follows: four had spinal cord injuries at or below the eighth thoracic vertebra, four had amputations, two had polio, one had cerebral palsy, and one had arthrogryposis. The participants mean age and body mass was 28.47 ± 5.79 years and 81.75 ± 17.33 kg, respectively. Body mass was assessed with specifically adapted equipment as described by Resende et al. [19].

The athletes participated voluntarily and signed an informed consent form in accordance with Resolution 466/2012 of the National Commission for Research Ethics (CONEP) of the National Health Council and the ethical principles of the latest version of the Declaration of Helsinki 2013 (and the World Medical Association). This study was approved by the Research Ethics Committee of the Federal University of Sergipe, CAAE: 2.637.882 (date of approval: 7 May 2018).

### 2.2. Experimental Design

The study comprised 3 weeks which included 9 sessions separated by a minimum of 48-h. The first three sessions (Week 1) were dedicated to baseline measurements of thermal images on Session 1, and to familiarization with the dynamic strength tests on Session 2 (1-RM and mean propulsive velocity) and the isometric strength tests on Session 3 (impulse, variability, peak torque). In Week 2 (Sessions 4 to 6), the participants performed in random order the three experimental conditions herein (3 types of warm-ups) followed 10-min later by the dynamic strength tests [21]. Skin temperature was measured immediately post-warm-up. In Week 3 (Sessions 7 to 9), the participants performed in random order the three experimental conditions herein (3 types of warm-ups) followed 10-min later by the isometric strength tests.

All testing was performed in an acclimated room, at the same time of day for and under the same environmental conditions (23 °C to 25 °C of temperature and relative humidity of ~60%). The athletes were asked to maintain the same routine during the evaluation days, avoiding strenuous exercise and refraining from consuming caffeine for 48-h before the test. This can be explained once caffeine tends to interfere in power, velocity, in static and fatigue states, which could interfere in the study’s results [22,23,24,25].

**Figure 1:**
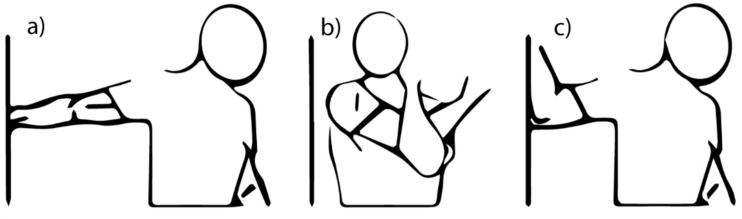
Stretches used as a warm-up method (**a**) shoulder, (**b**) triceps, and (**c**) pectoralis major.

The three types of exercise condition in terms of warm-up were: (i) exercise without any previous warm-up; (ii) exercise after traditional warm-up (which included dynamic resistance exercises); and (iii) exercise after a stretching warm-up (including 3 exercises as shown in Figure 1). A full explanation of the three types of war-up is described elsewhere [19,26].

#### 2.2.1. Stretching Warm-Up

The participants performed only three static stretching exercises for deltoids, chest, and triceps, as is shown in Figure 1 [26]. Stretching was performed gradually and slowly until the discomfort threshold at the subjective limit point and thus remained in the position for 30 s. The exercises were repeated 3 times with an interval of 10 s [26].

#### 2.2.2. Traditional Warm-Up

The participants performed the previous warm-up for the upper limbs, using three exercises (abduction of the shoulders with dumbbells, military press with dumbbells, and medial and lateral rotation of the arm to warm up the rotator cuff with dumbbells) with a set of 20 repetitions in approximately 10 min. Subsequently, a specific warm-up was performed on the bench press using only the barbell (20 kg) without extra weight, with 10 slow repetitions (3.0 × 1.0 s, eccentric × concentric) and 10 fast repetitions (1.0 × 1.0 s, eccentric × concentric). Next, the participants performed five repetitions at 30% of 1RM, followed by three repetitions at 50% of 1RM, a repetition at 70% of 1RM, a repetition at 80% of 1RM, and a repetition at 90% of 1RM. Between the series, the participants rested for 5 min [19,26].

### 2.3. Procedures

#### 2.3.1. Skin Temperature Measurement

Thermal image acquisition was performed in a room prepared without natural light, with no airflow directed to the collection site. Ambient temperature conditions were maintained at around 24 °C ± 1 °C, and relative humidity around 50% using an air conditioner and monitored by a hygrometer (HIGHMED, model HM-01, USA) [2].

Participants were instructed not to perform vigorous physical activity in the previous 24 h, not to consume alcohol or caffeine, and not to use any type of cream or lotion on the skin in the 6 h immediately prior to the evaluation. To obtain the thermograms, the athlete remained seated and did not make sudden movements, did not cross the arms, and did not scratch for a period of at least 10 min for acclimatization [2,27].

**Figure 2:**
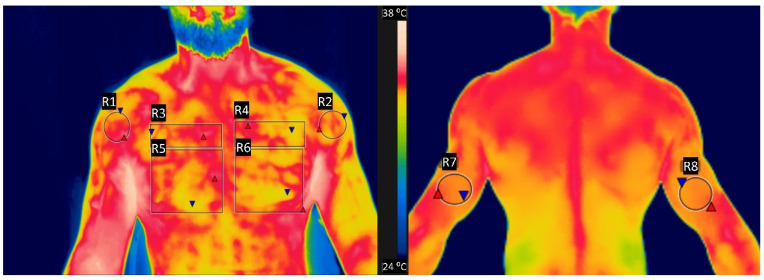
Illustration of the regions of interest (ROI) in the thermal images acquired.

Images were captured by an infrared camera model FLIR T640sc (Flir, Stockholm, Sweden) measuring range −40 °C to 2000 °C, accuracy 2%, sensitivity < 0.035, an infrared spectral band of 7.5–14 μm, refresh rate of 30 Hz, resolution of 640 × 480 pixels. The software used for thermal image analysis was FLIR TOOLS (Flir, Stockholm, Sweden). The region of interest evaluated was the anterior and posterior faces of the trunk and arms [2,28]. Figure 2 presents an illustration of the thermal images acquired.

#### 2.3.2. Dynamic Strength Measurements (1-RM and Mean Propulsive Velocity—MVP)

In every testing herein, a 2.1 m long official adapted bench (Eleiko Sport AB, Halmstad, Sweden), approved by the International Paralympic Committee [20], was used. The barbell was a 2.2 m long, 20 kg weight official bar (Eleiko Sport AB, Halmstad, Sweden). The 1-RM assessment in bench press exercise was performed following the protocol proposed by Fleck and Kraemer [29]. A 3.0 to 5.0 min rest was provided between attempts. A valid and reliable [30] linear position transducer (Chronojump, BoscoSystem, Barcelona, Spain) was attached to the bar to measure the velocity of movement. The maximum speed averages were collected with the 1-RM load [31].

#### 2.3.3. Isometric Force Measurements (Impulse, Variability, and Peak Torque)

The isometric evaluation was performed by having the participants press the bar at a distance of 15 cm from the chest. Impulse, variability, g and peak torque (PT) were measured with the help of a force sensor (Chronojump, BoscoSystem, Barcelona, Spain) and a goniometer FL6010 (Sanny, São José dos Campos, Brazil). Details of this testing can be found elsewhere [19].

### 2.4. Statistical Analysis

After confirmation of normality and homogeneity assumptions, one-way ANOVA with Bonferroni’s post hoc was performed to compare the measurements post- the three types of warm-up. A repeated-measures analysis of variance was used to evaluate the performance between the warm-up conditions, followed by Bonferroni post hoc comparison tests. To check the effect size, partial Eta squared (η2p) was used, adopting values of low effect (≤0.05), medium effect (0.05 to 0.25), high effect (0.25 to 0.50), and very high effect (>0.50) for ANOVA [32]. A d value <0.2 was considered a trivial effect, 0.2 to 0.6 a small effect, 0.6 to 1.2 a moderate effect, 1.2 to 2.0 a large effect, 2.0 to 4.0 a very large effect, and ≥4.0 an extremely large effect [33]. Cohen “d” was calculated as the difference between the mean divided by the pooled SD to estimate the effect size for between-lift comparison [32]. All statistical analyses were performed using the computerized package Statistical Package for the Social Science (SPSS), version 22.0 (IBM Corp, Armonk, NY, USA). The level of significance was set at *p* < 0.05. Data are presented as means (X) ± standard deviation (SD) and 95% confidence interval (95% CI).

## 3. Results

The participants’ 1-RM in bench press was 119.07 ± 43.15, which corresponded to a mean of 1.50 ± 0.38 times their body mass. Values above 1.4 (1RM/body weight) in the bench press, are considered to classify elite athletes [34].

The results found in the dynamic indicators of force, MPV, and power, and static indicators of force, impulse, and variability in relation to the different types of warm-up are shown in Table 1.

**Figure 3:**
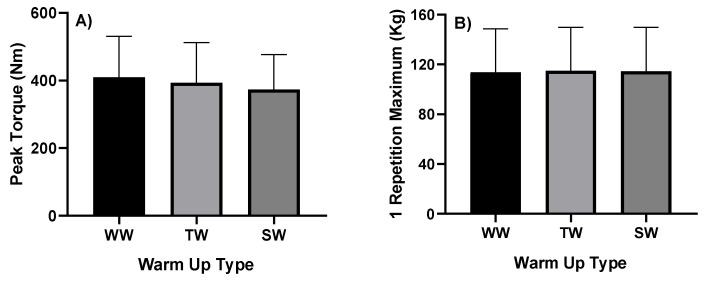
Evaluation of (**A**) peak torque (N.m) and (**B**) 1 repetition maximum (Kg), with different types of warm-up. WW: without warm-up, TW: traditional warm-up, and SW: stretching warm-up.

Table 2 shows no differences in the dynamic and static indicators of the force in relation to the different types of warm-up. The results found for peak torque (Nm) and 1 maximum repetition (kg) are shown in Figure 3.

No significant differences were found in relation to the (A) peak torque (*p* = 0.055, F = 4.560, η2p = 0.246 medium effect), and (B) 1 repetition maximum (*p* = 0.139, F = 3.191, η2p = 0.186, medium effect) between the different types of warm-up.

## 4. Discussion

The objective of our study was to evaluate the different types of warm-up: without warm-up, traditional warm-up, and warm-up with stretching, on thermographic and strength indicators. The results of the mean propulsive velocity (MPV) did not show differences between the types of warm-up, however, the condition without warm-up (WW) was the one that presented the highest average propulsive speed (1.12 ± 0.06 m/s), followed by traditional warm-up (TW) (0.11 ± 0.05 m/s), and warm-up with stretching (SW) (0.10 ± 0.04 m/s). 

A study that evaluated the influence of specific warm-up on strength performance found that participants were able to achieve a higher propulsive speed in the second and third sets in the squat, and with a tendency to decrease propulsive speed in the bench press. The time for propulsive speed was shorter after a warm-up with progressive intensity, demonstrating that speed can be affected by warm-up, tending to decrease with more activities [7]. In the horizontal bench press, the MPV does not tend to be greater at the beginning of the training, which is explained by the lower muscle mass involved, when compared to other exercises, in addition to being a relatively simple movement [35]. Thus, it seems that a more relaxed muscle tends to establish a higher speed than after a more traditional warm-up. The reduction in movement speed during strength work tends to indicate fatigue [14,36]. On the other hand, stretching exercises for sports training [37] and for maximum tests or for competition are highly questionable and would normally be related to loss of performance [38]. Still, the elapsed time of the warm-up with the use of stretching must observe an interval greater than three minutes before the warm-up continues [26].

Concerning power, despite not showing differences between the three types of warm-ups, the condition without warm-up showed a higher power (137.69 ± 90.80 W), followed by traditional warm-up (128.86 ± 69.70 W), and warm-up with stretching (105.77 ± 50.77 W). If we consider that power is the product of strength times speed, speed in resistance training can be considered very important when assessing muscle strength [39,40]. Whether traditional or even with pre-activation, the warm-up aims to increase the muscle temperature, the activation of the motor unit, and the myofiber water content [41,42]. Moderate to heavy exercises with loads varying between 20–90% of a maximum repetition tend to improve sprint and jump, especially in participants trained and familiar with the exercise load [43,44]. However, our findings indicate that WW and TW tend to be better than SW. On the other hand, contrary to this, dynamic and static stretching tend to be favorable as a warm-up strategy [12,26]. In active individuals, dynamic stretching increased the height of the vertical jump. On the other hand, agility tends to be positively impacted by stretching. Dynamic stretching can improve an athlete’s power [45].

The same kinetics occurred in relation to the static components of the force, where there were no differences in impulse, and the WW method obtained the highest value (4022.23 ± 1341.43 N.s), followed by TW (3964.91 ± 1240.10 N.s) and of the SW (3740.41 ± 1114.96 N.s). In the same direction, the participants tested after 6.0 min of swimming warm-up or warm-up on land, with three repetitions (pull-over at 85% of the maximum of one repetition). Speed, force, acceleration, impulse, rate of force development (RFD) were evaluated. Warm-up on land with higher loads increased RFD (34.52 ± 16.55 vs. 31.29 ± 13.70 N/s; Δ = 9.35%), and stroke rate (64.70 ± 9.84 vs. 61.56 ± 7.07 Hz; Δ = 5.10%) compared to traditional water warm-up but decreased speed, strength, acceleration, impulse and power [46]. That is, traditional warm-up can decrease the impulse, and a lighter warm-up tends not to decrease the impulse. Likewise, exercises with high load resistance have been used to facilitate the improvement of neuromuscular performance. Traditional warm-up has its use restricted, despite its specificity and practicality for sports performance. Thus, when verifying the effect of repeated exercises on performance, where 43 participants were evaluated performance was quantified through vertical jump, relative thrust, and normalized peak strength at baseline. No improvements were found for the relative impulse in repeated trials, the sixth trial was significantly less than the baseline (2.35 ± 0.38 vs. 2.26 ± 0.35 N·s·kg; *p* ≤ 0.001). This indicates that the repetition of traditional warm-ups can lead to fatigue, which tends to interfere with performance [47].

In variability, although there are no differences between the types of warm-up, the least variability was TW (40.57 ± 17.72 N), followed by SW (41.26 ± 23.42 N) and WW (46.74 ± 30.06 N). This may indicate that traditional warm-up tends to promote a more stable situation on a muscular level than other warm-up types, noting that there were no significant differences between the warm-up methods.

There were also no differences in peak torque, however, the WW method showed higher values (409.58 ± 120.99 Nm), followed by TW (393.74 ± 118.48 Nm) and SW (373.14 ± 103.51 Nm). One study evaluated standard warm-up or drop jump (plyometric protocol) or a slow walk (control protocol). Post-activation potentiation was assessed by changes in isometric muscle contractions. The plyometric protocol increased the peak contraction torque (PTT), the rate of torque development (RTD) and the impulse significantly (by 23, 39 and 46%, respectively). Peak contraction torque, RTD, and impulse, decreased significantly after standard warm-up. Thus, standard warming did not enhance but may have reduced the muscle’s ability to generate strength [48]. The data in this study contradict our findings.

In the 1RM test, the three methods also showed no differences, however, the TW method (114.80 ± 34.98 kg) showed higher values, followed by the SW (114.53 ± 35.20 kg) and the WW (113.80 ± 34.80 kg). A specific warm-up can increase the production of strength after maximum or almost maximum muscle stimulation [14]. The effects of warming on athletic success have gained great attention in recent studies. Authors [19] evaluated different types of warm-up, with the participation of 15 elite Brazilian male athletes from Paralympic powerlifting (age, 24.140 ± 6.21 years; body weight, 81.67 ± 17.36 kg). A significant difference was observed for the maximum isometric strength, in the without warm-up (WW) in relation to traditional warm-up (TW) and stretching warm-up (SW) (*p* = 0.005, η2p = 0.454, high effect). On the other side, no significant differences were observed in the RFD, fatigue index (FI), and time in the different types of warm-up (*p* > 0.05). No significant differences were observed in relation to the maximum repetition (*p* = 0.121, η2p = 0.275, medium effect) or the maximum speed (*p* = 0.712, η2p = 0.033, low effect) between the different types of warm-up. The different warm-up methods do not seem to provide significant differences in strength indicators in this population, and this could be explained by the displacement they use to the upper limbs, the target of the study [19].

In the thermographic analysis, there was a significant difference only in the pectoral muscle clavicular portion between the TW (33.04 ± 0.71 °C) and the WW (32.51 ± 0.74 °C) (*p* = 0.038). The TW method also presented slightly higher values than the SW and WW in the pectoral muscles sternal portion and in the deltoid anterior portion, but with a *p*-value > 0.05. These results are in agreement with authors who studied thermal response to resistance training [49]; since traditional warm-up involves specific resistance exercises for the primary muscles that are recruited in the main work.

The physiological reason for the increase in skin temperatures observed in the pectoral muscle clavicular portion (TW protocol) might be the increase in the recruitment of motor units, which occurs during the traditional warm-up, needed to prepare the muscles for the considerable effort needed to overcome the weight of the barbell and give it acceleration [50].

Authors [49] reported an increase in skin temperature over the muscles that were the main responsible for the movement requested after the exercise. Neves et al. [28] reported that the warming of ROI in arm exercise seems to be related to exercise volume. In this sense, since the traditional warm-up protocol included a large volume of exercises, it promoted an increase in blood flow to the pectoral muscle clavicular portion and, consequently, a greater heat dissipation by the skin over this muscle.

From the hypotheses raised, our findings, and the confrontation with the literature, we ended up supporting the idea that, for Paralympic athletes, the type of warm-up tends not to impact performance in terms of temperature and strength indicators.

The study used the functional classification adopted by the International Paralympic Committee. Thus, it can be mentioned as limitations of this study, the control of variables such as balance, food, and life habits.

## 5. Conclusions

It can be concluded that the type of warm-up does not seem to interfere with the performance of Paralympic powerlifting athletes. However, although there are no significant differences between the warm-up methods, the thermal images showed that traditional warm-up best meets the objectives expected for this preparation phase. In a competition, it could be enough to provide better performance and classification.

The results found may have been influenced by the condition of being in a wheelchair and requiring the use of the upper limbs and trunk muscles for displacement, thus promoting the maintenance of these muscle groups in a state of activity similar to what is observed after the warm-up protocols.

Another important point is that in high-level competitions and especially in the Paralympic Games, many weight categories were classified by results with differences of less than 5.0%. In this sense, in competitions, when determining the type and warm-up, the traditional would be the best indicated in high-level competitions and, in training, any type of warm-up could be used.

## Figures and Tables

**Table 1:** Dynamic and static force indicators (mean ± standard deviation, 95% CI) in relation to different types of warm-up.

Warm-Up	MPV (m/s) X ± DP (IC 95%)	Power (W) X ± DP (IC 95%)	Impulse (N.s) X ± DP (IC 95%)	Variability (N) X ± DP (IC 95%)
Without Warm-up (A)	0.12 ± 0.06 (0.09–0.16)	137.69 ± 90.80 (87.41–187.97)	4022.23 ± 1341.43 (3279.36–4765.09)	46.74 ± 30.06 (30.10–63.38)
Traditional (B)	0.11 ± 0.05 (0.09–0.14)	128.86 ± 69.70 (90.26–167.46)	3964.91 ± 1240.10 (3278.17–4651.66)	40.57 ± 17.72 (30.76–50.38)
Stretching (C)	0.10 ± 0.04 (0.07–0.12)	105.77 ± 50.77 (77.65–133.88)	3740.41 ± 1114.96 (3122.96–4357.85)	41.26 ± 23.42 (28.29–54.22)
A vs. B	*p* = 0.62 d = 0.18	*p* = 0.77 d = 0.11	*p* = 0.90 d = 0.04	*p* = 0.50 d = 0.25
A vs. C	*p* = 0.29 d = 0.39	*p* = 0.25 d = 0.43	*p* = 0.54 d = 0.23	*p* = 0.58 d = 0.20
B vs. C	*p* = 0.55 d = 0.22	*p* = 0.31 d = 0.38	*p* = 0.61 d = 0.19	*p* = 0.93 d = 0.03
*p*	0.272	0.383	0.293	0.999
η2p	0.122 ^##^	0.116 ^##^	0.121 ^##^	0.030 ^#^

*p* < 0.05 (ANOVA two-way, and Bonferroni post hoc, ES η2p). A vs. B; A vs. C and B vs. C (test t and ES Cohen “d”). ES: effect size, ^#^ low effect, ^##^ medium effect. MPV: mean propulsive velocity.

**Table 2:** Skin temperature over active muscles in different types of warm-up (mean ± SD and CI 95%).

Muscles	WW (°C)	TW (°C)	SW (°C)	WW vs. TW	WW vs. SW	TW vs. SW	*p*-Value	η2p
Pectoral Sternal	31.77 ± 1.18 (31.11–32.42)	32.23 ± 0.80 (31.79–32.68)	32.06 ± 1.09 (31.45–32.67)	*p* = 0.238 d = 0.456	*p* = 0.505 d = 0.255	*p* = 0.642 d = 0.178	0.261	0.091 a
Pectoral Clavicular	32.51 ± 0.74 * (32.10–32.92)	33.04 ± 0.71 * (32.65–33.43)	33.02 ± 0.55 (32.72–33.32)	*p* = 0.064 d = 0.731	*p* = 0.049 * d = 0.782	*p* = 0.934 d = 0.031	0.038 *	0.276 b
Anterior Deltoid	32.31 ± 0.69 (31.93–32.69)	32.52 ± 0.78 (32.09–32.95)	32.64 ± 0.60 (32.31–32.97)	*p* = 0.457 d = 0.285	*p* = 0.230 d = 0.465	*p* = 0.061 d = 0.741	0.145	0.099 a
Triceps	31.47 ± 0.93 (30.96–31.99)	31.99 ± 0.54 (31.70–32.29)	32.01 ± 0.80 (31.57–32.46)	*p* = 0.082 d = 0.684	*p* = 0.112 d = 0.623	*p* = 0.939 d = 0.029	0.108	0.193 a

* *p* < 0.05 (ANOVA). (WW in comparison with TW). η2p = partial eta square. “a”: medium effect and “b”: high effect. d: Cohen’ d. WW: without warm-up, TW: traditional warm-up and SW: stretching warm-up.

## Data Availability

The data that support this study can be obtained from the address https://www.asuswebstorage.com, accessed on 1 July 2021.
